# Burden of heat stress on residual work capacity among farmers living with chronic HIV in Siaya county, Kenya: a longitudinal observational study protocol

**DOI:** 10.1186/s12889-025-24373-w

**Published:** 2025-08-27

**Authors:** Daniel Kwaro, Nour Kassem, Stephen Munga, Julius Okoth, Hanns-Christian Gunga, Sandra Barteit, Martina Anna Maggioni

**Affiliations:** 1 Institute of Physiology, Center for Space Medicine and Extreme Environments, Charité – Universitätsmedizin Berlin, Berlin, Germany; 2 Kenya Medical Research Institute, Kenya, Kenya; 3 Institute of Global Health (HIGH), Heidelberg University Hospital, Heidelberg University, Heidelberg, Germany; 4 Department of Biomedical Sciences for Health, Università degli Studi di Milano, Milan, Italy

**Keywords:** Extreme heat, Heat strain, Work capacity, Sleep quality, Farmers, HIV infection, Occupational health, Africa

## Abstract

**Introduction:**

Sub-Saharan Africa, including Siaya County in Kenya, has a high prevalence of chronic HIV infection, which may increase vulnerability to climate-induced heat stress among agricultural workers. Understanding how HIV moderates the relationship between environmental heat exposure and labour capacity is essential for designing targeted, equitable public health interventions in climate-vulnerable settings. This study aims to quantify the effects of heat exposure on labour capacity and sleep, assess whether physiological strain mediates these effects, and examine whether HIV status and sex affect the observed relationships.

**Methods:**

This is an ongoing 24-month longitudinal observational study involving 124 participants (62 male–female pairs) stratified by HIV status. HIV-positive participants are recruited from the Wagai Health Centre’s HIV clinic, and HIV-negative participants are recruited from the general population via the Siaya County Health and Demographic Surveillance System (HDSS) registry. The participants are aged 20–45 years and engaged in agricultural livelihoods. Environmental heat exposure (indoor and outdoor wet bulb globe temperature), actigraphy (physical activity and sleep), and physiological metrics (heart rate and core body temperature) are continuously or periodically monitored using research-grade wearables. GPS data and monthly questionnaires on thermal comfort, work timing, and heat-related symptoms are collected to contextualize physiological responses. Data collection occurs sequentially—first 12 months for the HIV-negative group and then another 12-months for the HIV-positive group—ensuring seasonal alignment across cohorts. Mixed-effects models will assess the associations between heat exposure and residual labour capacity (primary outcome) and sleep quality (secondary outcome), examining mediation by physiological strain and moderation by HIV status and sex. The models will be adjusted for age, body composition, and other potential confounders.

**Discussion:**

This study will generate novel evidence on the impact of heat stress on labour capacity and sleep in HIV-positive populations, addressing a critical gap in climate-health research in sub-Saharan Africa. Findings will inform equitable adaptation strategies, such as work-rest cycles and hydration protocols, tailored to vulnerable subgroups, including women and individuals living with HIV.

**Supplementary Information:**

The online version contains supplementary material available at 10.1186/s12889-025-24373-w.

## Introduction

By the end of the century, sub-Saharan Africa (SSA) is expected to experience rapid warming, with mean annual temperatures projected to rise by 1.4 °C in low-emission scenarios and up to 4.4 °C in high-emission scenarios [1]. Productivity losses are already evident and are often attributed to heat-induced crop failure and livestock stress, trends that are expected to intensify as temperatures rise [1, 2]. In addition to these impacts on crops and animals, emerging empirical evidence, primarily from outside SSA, highlights a second critical pathway: a reduced ability to perform physical work due to the physiological impact of working in high heat [1, 2].

This mechanism may be particularly consequential in SSA, where more than 50% of the rural population engages in manual, outdoor farming [3]. These farmers often work in high ambient temperatures with limited access to shade, cooling, or hydration infrastructure [4]. Despite this high-risk context, empirical studies quantifying heat-related labour loss in SSA remain limited, constraining the development of effective adaptation strategies and occupational health policies [5].

### Physiological basis of heat strain

Understanding how heat stress affects human physiology is essential for interpreting its impact on labour capacity, particularly among individuals with chronic illness. Heat stress is the thermal load that the body’s thermoregulatory system must dissipate to maintain a stable core body temperature (CBT) of approximately 37 °C (± 0.5 °C). It can be endogenous, generated internally, for example, during physical activity, or exogenous, arising from external factors such as high ambient temperatures [6, 7]. The body’s thermoregulatory response to rising heat is coordinated through an integrated physiological network [7–10]. Thermoreceptors in the skin and core detect changes in temperature and signal the preoptic area of the hypothalamus, which activates the autonomic nervous system (ANS). This initiates cutaneous vasodilation to increase skin blood flow and sweating, thus aiding cooling via convective and evaporative heat loss, respectively. The cardiovascular system increases the heart rate (HR) and redistributes blood to the periphery. Baroreceptors detect central blood volume shifts caused by peripheral pooling and trigger reflex sympathetic activation to maintain blood pressure.

Additional systems help preserve homeostasis. The renal and endocrine systems preserve plasma volume via aldosterone and vasopressin, and thyroid and metabolic hormones adjust energy expenditure [11, 12]. At the cellular level, heat shock proteins (HSPs) are upregulated in response to rising core temperature, helping maintain protein integrity and modulating inflammation, thereby supporting thermal tolerance [13]. Alongside these physiological and cellular processes, the brain also initiates behavioural adaptations, including reducing physical activity, seeking shade, removing excess clothing, or increasing fluid intake [7]. When heat exposure is extreme or physiological systems are impaired, these mechanisms may be insufficient. As a result, the core temperature and heart rate rise, leading to physiological strain - a cumulative internal stress response to heat [2, 7, 14].

Physiological strain can be quantified using the Physiological Strain Index (PSI), which compares CBT and HR against baseline and critical values on a scale of 0–10 [14]. Variants include the adaptive PSI (aPSI), which adjusts skin-to-core temperature gradients [15], and the modified PSI (mPSI), which is adapted for field use [16]. PSI has also been integrated with accelerometer data to form the Physiological and Activity Strain Index (PASI), which captures both physiological strain and activity-related risks [17].

### Mediating role of heat strain in heat-induced loss in work capacity

To capture environmental heat stress, the Wet Bulb Globe Temperature (WBGT) index is commonly used. WBGT integrates the air temperature, humidity, solar radiation, and wind speed to estimate the external thermal burden [18]. Numerous studies consistently show a strong correlation between increasing WBGT and reductions in labour capacity [19–21].

Labour capacity refers to the maximum potential of an individual to perform physical work, given their physiological and environmental conditions. Under heat stress, individuals often experience a decline in residual labour capacity, the portion of labour capacity that remains after accounting for factors that determine individual thermoregulatory efficiency, such as age, sex, body composition, fitness level, and acclimatization [1, 2]. Labour performance, on the other hand, is the actual amount of physical work carried out, which also declines [1, 2, 22, 23]. A meta-analysis by Flouris et al. (2018) estimated that under heat stress (WBGT beyond 22.0 or 24.8 °C— depending on work intensity), 35% of workers experienced heat strain, and 30% reported productivity losses [2]. This is because, as physiological strain increases in response to higher WBGT levels, workers must reduce efforts to prevent heat-related illnesses (HRIs), such as heat exhaustion and heatstroke [14, 16, 24, 25]. Such coping strategies may include lowering work intensity, taking more frequent breaks, or rescheduling tasks to cooler hours. For example, Yengoh et al. (2020) estimated that in eastern and coastal Kenya and Tanzania, projected temperatures in February and March could require up to 75% rest per hour for safe work in 2050 and 2100 [26]. While essential for safety, these behavioural adaptations come at the cost of reduced work performance.

Beyond its impact on physical performance, heat stress affects health and well-being more broadly, contributing to sleep disruption and cognitive impairments that increase the risk of errors and poor decision-making in the workplace [27, 28]. In addition, heat stress can exacerbate preexisting cardiovascular and respiratory conditions, increase the risk of dehydration and kidney injury, and negatively impact mental health [7, 29, 30]. Sleep disruption itself may also contribute to the cumulative burden of chronic diseases, particularly among vulnerable populations [27].

### Chronic HIV infection and thermoregulatory vulnerability

Chronic illnesses can further heighten susceptibility to heat-related strain by impairing various components of the thermoregulatory system. For example, conditions such as diabetes mellitus [31], chronic kidney disease (CKD) [11], and cardiovascular disease (CVD) [32] reduce heat tolerance by disrupting vasodilation, the sweat response, fluid regulation, and cardiac output. These impairments have been associated with increased physiological strain and diminished work performance [2, 7], although most of the available evidence is from high-income countries, yet their burden is rising in SSA [33].

**Fig. 1:**
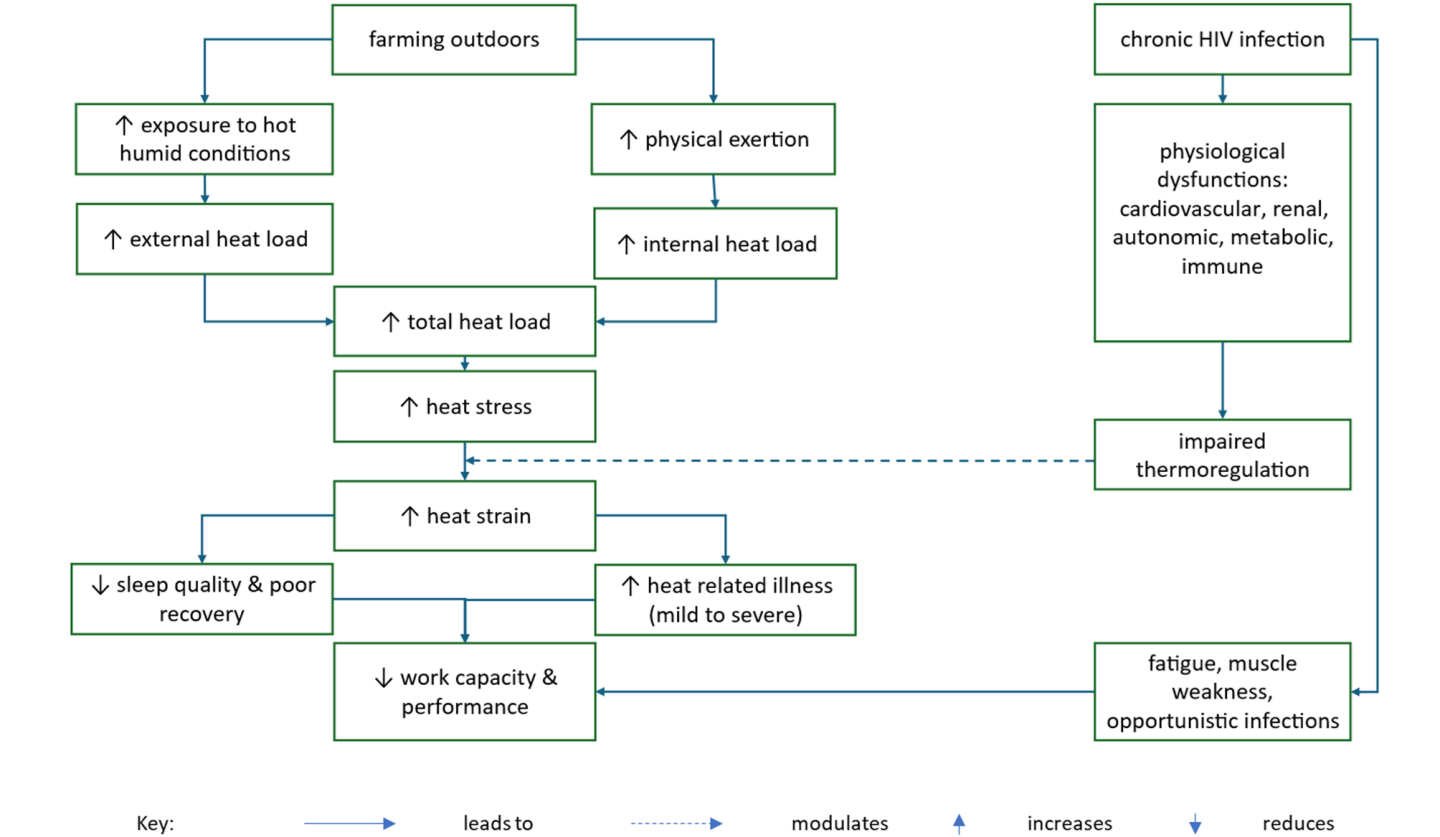
Conceptual framework illustrating the impact of extreme heat on work performance in HIV-positive farmers. This diagram illustrates how farming under hot, humid conditions increases both external and internal heat loads, culminating in total heat strain. In HIV-positive individuals, chronic infection further modifies this pathway by reducing thermoregulatory tolerance, thereby indirectly diminishing work performance. Moreover, HIV can directly impair physical capacity through mechanisms such as fatigue, muscle weakness, and heightened susceptibility to opportunistic infections

HIV, while now a chronic condition due to widespread access to antiretroviral therapy (ART), remains highly prevalent in SSA [34]. However, its implications for thermoregulation and heat-related work performance are poorly understood. Like other chronic diseases, HIV affects multiple systems critical for thermal homeostasis (Fig. 1). For example, the cardiovascular system is impacted by HIV-associated endothelial dysfunction and accelerated atherosclerosis [35–37], which directly impair cutaneous vasodilation and limit cardiovascular reserve, which is critical for dissipating heat via convection and evaporation mechanisms [10]. Moreover, autonomic dysfunction in HIV-positive individuals is characterized by reduced heart rate variability and impaired baroreflex sensitivity [38], which can compromise sweating responses and blood pressure regulation during heat exposure. Additionally, HIV-associated nephropathy significantly impairs fluid regulation capabilities [39–41], likely exacerbating dehydration risk under prolonged heat exposure. Immunologically, persistent immune activation in HIV, even among individuals receiving effective antiretroviral therapy, promotes increased DNA methylation and accelerated biological aging [42–45], potentially reducing physiological reserves across multiple organs, including the cardiovascular and renal systems [44, 46, 47]. This chronic inflammatory state may further induce a prothrombotic environment, impairing circulation critical to heat dissipation [44, 48]. Metabolically, complications associated with chronic HIV infection, such as lipodystrophy due to antiretroviral therapy , diabetes, and thyroid disorders, disrupt fat distribution and energy metabolism [46, 49–51]. These alterations in body composition and metabolic function could reduce thermal insulation efficiency and impair the metabolic energy needed for effective thermoregulatory responses [7]. Finally, evidence suggests that heat exposure itself directly enhances HIV virus replication rates in CD4 + T cells [52]. This may accelerate CD4 + T-cell depletion and compromise immune regulation of inflammatory responses that affect heat dissipation.

HIV may also impair work performance and sleep quality through nonthermal pathways. Chronic inflammation, immune dysregulation, and neurocognitive disorders can disrupt sleep architecture, whereas pain, anxiety, and side effects of ART may further reduce sleep efficiency and increase fatigue [53]. Poor sleep impairs overnight recovery, lowers physiological reserves, and heightens vulnerability to thermal strain [27, 54, 55]. Additionally, other extreme weather events such as flooding and storms often disrupt health services, leading to treatment interruptions, opportunistic infections, and mental health challenges—all of which contribute to absenteeism and reduced productivity [56].

Despite these plausible mechanisms, no empirical studies to date have tested whether HIV moderates the effects of heat exposure on physiological strain or work performance. Given the high prevalence of HIV in SSA and the region’s intense exposure to climate extremes, this represents a critical knowledge gap for climate-health adaptation efforts.

### Research gaps and study objectives

Most existing evidence on heat intolerance comes from studies in healthy adults or individuals with noncommunicable diseases. We do not know whether people living with HIV (PLWH) exhibit steeper increases in physiological strain or greater declines in physical activity and sleep quality at equivalent heat exposure levels. Nor is it clear whether these effects are fully mediated through thermoregulatory strain, or if HIV-specific mechanisms act independently.

This study addresses these gaps by testing three hypotheses among rural Kenyan farmers:

1. Higher WBGT is associated with reduced physical activity (primary outcome), a proxy for work capacity, and poorer sleep quality (secondary outcome).
2. HIV infection amplifies these effects.
3. Physiological strain mediates the relationship between heat exposure and these functional outcomes (activity and sleep), with this mediation modified by HIV and individual-level factors such as sex, age, and body composition.

We use research-grade wearable sensors to continuously monitor CBT, heart rate, physical activity (e.g., steps, time spent in moderate-to-vigorous activity), and sleep parameters (e.g., duration, efficiency, onset latency) in HIV-positive and HIV-negative farmers. These data will be integrated with environmental measurements of WBGT from an automatic weather station and indoor data loggers, and comparisons between the two groups will be made. Unlike self-reports or spot measurements, this continuous, high-resolution, non-invasive approach improves ecological validity and sensitivity [57–60]. The feasibility of this method in rural SSA was established in our prior Kenyan study [61].

Women, who constitute a substantial share of SSA’s agricultural workforce but remain underrepresented in occupational health research, are also included [62]. By integrating physiological, behavioural, and environmental data, this study provides new insights into how HIV may shape vulnerability to heat stress, informing climate-health adaptation strategies for high-risk populations in low-resource settings.

## Materials and methods

### Study design

This ongoing longitudinal, mixed-methods cohort study assesses the impact of environmental heat stress on residual labour capacity among agricultural workers in Siaya County, Kenya. Two cohorts will be followed over consecutive 12-month periods: HIV-negative participants in Year 1 (2022/2023) and HIV-positive participants in Year 2 (2024/2025). The sequential design allows for cost-effective reuse of research-grade wearable devices. To minimize the risk of temporal confounding, each cohort is followed during the same calendar months, enabling the alignment of seasonal trends in heat exposure and agricultural activity. Any residual differences in environmental or socioeconomic conditions across years will be accounted for analytically. Data collection integrates personal and environmental monitoring (e.g., heart rate, core body temperature, physical activity, and indoor and outdoor WBGT), structured questionnaires, and anthropometric measurements.

### Study setting and population

**Fig. 2:**
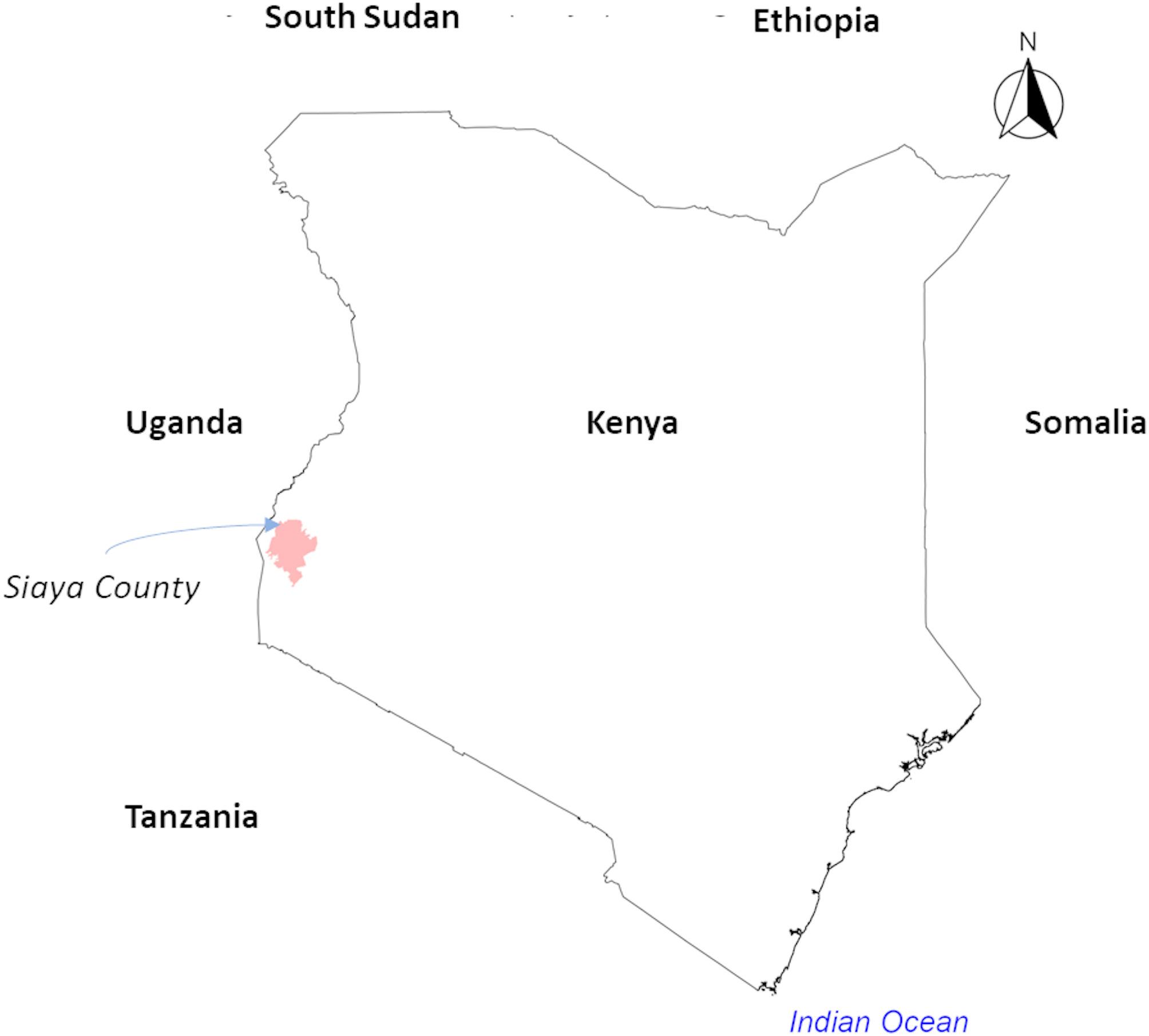
Location of the study area in Kenya. Map highlighting the study site in red, located in Siaya County, western Kenya

This study is being conducted in rural agricultural communities within Siaya County, western Kenya (Fig. 2), a region heavily reliant on subsistence farming and with a high HIV prevalence of approximately 21%, whereas the national average is approximately 4.9% [63, 64]. The study area includes a 10-kilometer radius around Wagai Village in Gem subcounty, within the KEMRI/CDC Health and Demographic Surveillance System (HDSS) [65].

Wagai lies within the Tropical Savanna (Aw) climate zone, characterized by distinct wet and dry seasons [66–69]. The annual rainfall ranges from 900 to 1,200 mm, with two rainy seasons occurring in March–May and October–December. The elevation in the area ranges from 1,200 to 1,300 m above sea level, contributing to moderate-to-high humidity. Ambient daytime temperatures typically range from 20 to 30 °C, frequently exceeding physiological comfort thresholds during peak heat hours.

The study area falls within the lower midland agroecological zones (LM3–LM5) [68, 69], where households engage in labour-intensive cultivation of crops such as maize, beans, cassava, and sorghum. Agricultural workers perform prolonged moderate-to-high intensity tasks in unshaded, outdoor environments, increasing the risk of physiological heat strain, reducing work capacity, and impacting occupational productivity. This setting presents a highly relevant context for examining how chronic HIV infection may interact with environmental heat stress to affect health and labour outcomes.

### Eligibility criteria

Participants are enrolled as cohabiting male–female pairs (spouses or long-term partners) to:

- Examine sex-based differences in physiological responses to heat stress under comparable exposures,
- Assess gendered patterns in device use and daily routines, and.
- Minimize cultural barriers to female participation through joint enrolment.

All participants must meet general inclusion criteria, with additional requirements for the HIV cohort. Criteria are designed to support safety, data quality, and retention across the 12-month follow-up period.

### Inclusion criteria

*All participants must:*

- Be aged 20–45 years at screening.
- Be married or cohabiting with their study partner.
- Have resided in the HDSS area for ≥ 4 months (excluding temporary/rental housing).
- Rely on agriculture as the primary livelihood (self or partner).
- Be registered in the HDSS for demographic linkage and follow-up.
- Be fluent in English, Kiswahili, or Dholuo.

*HIV cohort only:*

- Have a documented HIV diagnosis and ≥ 12 months in HIV care.
- Have disclosed HIV status to their partner.

### Exclusion criteria

*Participants are excluded if they:*

- Have acute illness, disability, or medical conditions that limit physical activity.
- Have a diagnosis, other than HIV, affecting thermoregulation (e.g., CVD, diabetes, COPD).
- Are currently pregnant.
- Have a BMI > 25 kg/m².
- Have experienced serious alcohol-related health or social issues in the past year.
- Plan to migrate from the HDSS area during the study period.
- Are unable or unwilling to provide informed consent.

### Sampling and sample size

#### Sampling methods

Participants are recruited using random sampling from two distinct registries. For the HIV-negative cohort, households containing cohabiting male–female pairs within the target age range (20–45 years) are randomly selected from the HDSS database. For the HIV-positive cohort, individuals are sampled from the HIV clinic registry at Wagai Health Centre, including only those enrolled in care for at least 12 months. Each selected individual is paired with their spouse if the spouse is also registered at the same clinic. If not, the individual is asked to refer their partner, provided that the partner has been living with HIV for more than one year.

#### Sample size justification

Sample size estimation was based on the primary physiological outcome of peak heart rate (HR) during physical activity, a well-established marker of cardiovascular strain under heat stress. In a comparable field study, an ambient air-temperature increase of 6 °C (from 28 –30 °C to 35–36 °C) was associated with an increase in peak HR of approximately 10 beats-per-minute (bpm) —from 119 ± 10.5 to 132 ± 11.5 bpm— corresponding to a large effect size (Cohen’s d ≈ 1.18) [70]. For conservative planning, we assumed a moderate effect size (d = 0.5).

Using a two-sided paired t-test with 80% power, α = 0.05, and an intraindividual correlation of *r* = 0.5 across repeated seasonal measures, a minimum of 35 participants would be required to detect such an effect. However, adjustments were made for the following design features:

- Repeated measures: Each participant contributes 12 monthly observations. Although repeated measurements increase power when analyzed using mixed-effects models, the base sample size was kept conservative to ensure robustness.
- Household clustering: Participants are recruited as cohabiting male–female pairs, introducing mild clustering. Assuming a household-level intraclass correlation (ICC) of 0.1 with a cluster size of 2, the design effect is modest (DE = 1.1) but was considered in planning.
- Stratification by HIV status and sex: Subgroup analyses are planned to compare outcomes by HIV status and sex. Ensuring ≥ 30 participants per subgroup (e.g., HIV-positive women) allows sufficient power for stratified analysis.
- Anticipated attrition: A 12-month follow-up period may result in up to 15% attrition due to migration or non-adherence. The target sample was inflated accordingly.

On the basis of these considerations, we aim to recruit 60 participants per cohort (HIV-positive and HIV-negative), equally split by sex (30 males, 30 females), for a total of 120 participants. This sample size provides adequate power to detect moderate effects in peak HR and related outcomes, while supporting stratified and longitudinal analysis using linear mixed-effects models, with participant and household specified as random effects and time, season, and HIV status as fixed effects.

### Recruitment, screening and enrolment

**Fig. 3:**
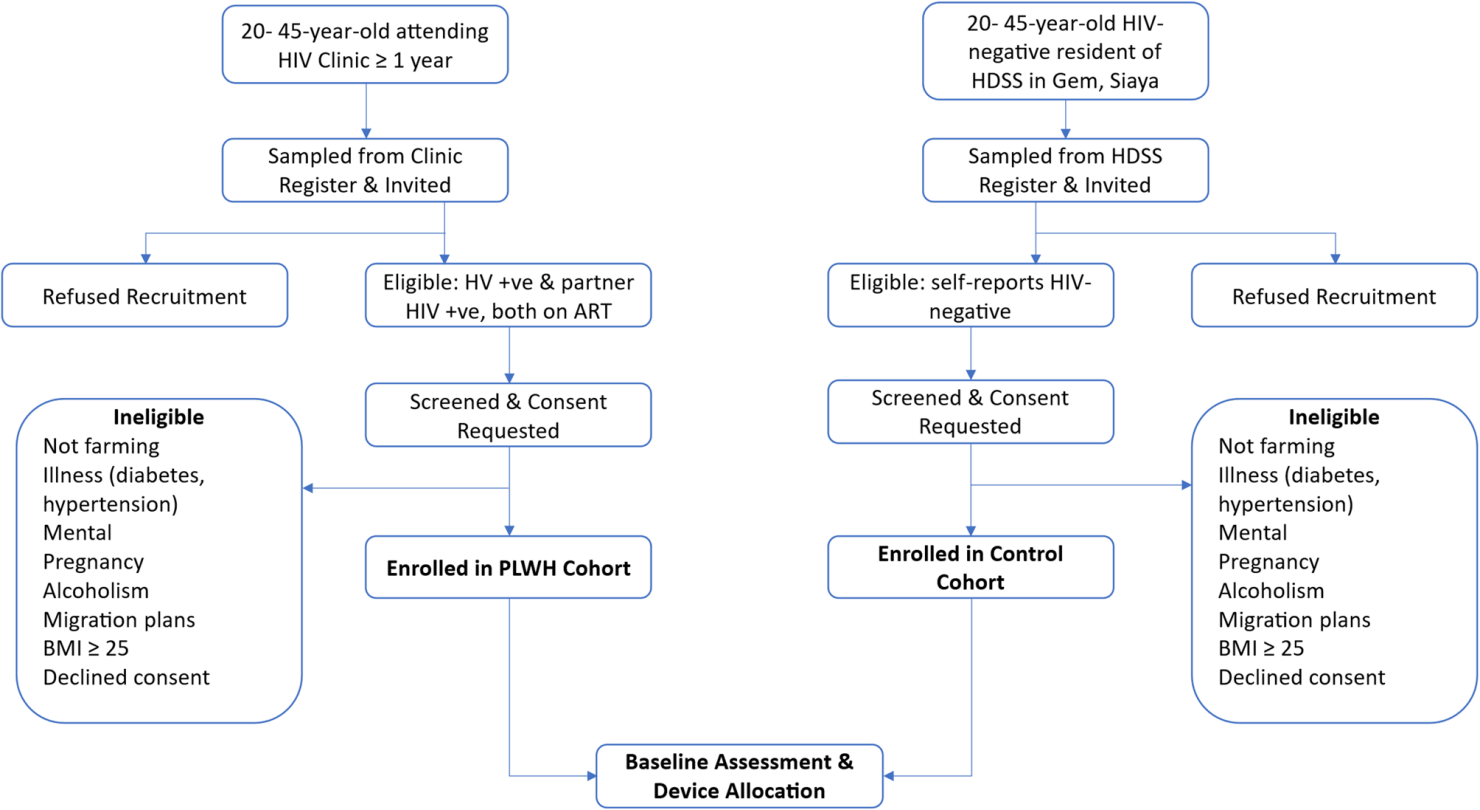
Recruitment and enrollment flow for study cohorts. Flow chart showing the recruitment, screening, and enrollment of HIV-positive and HIV-negative cohabiting adults in Siaya, Kenya. Eligible participants met health, residency, and consent criteria and were allocated to PLWH (Persons Living with HIV) or control (General Population, HIV-negative) cohorts for baseline assessment

Participants are recruited through two context-specific approaches tailored to each study cohort (Fig. 3).

For the HIV-positive cohort, eligible individuals are identified from the HIV clinic registry at the Wagai Health Centre. Recruitment is conducted during routine clinic visits, where participants are approached by trained study staff in private, confidential settings. Individuals who meet eligibility criteria are invited to enroll along with their cohabiting partner, provided that the partner is also HIV-positive and has been diagnosed for at least 12 months. If the partner is not present, the index participant is encouraged to invite them for screening. If the partner is unwilling or ineligible, the pair is excluded to maintain the dyadic study design. Care is taken to prevent inadvertent disclosure of HIV status, and all consent procedures are conducted with strict attention to privacy and confidentiality.

For the HIV-negative cohort, recruitment is conducted through the KEMRI/CDC Health and Demographic Surveillance System (HDSS). Trained fieldworkers carry out door-to-door outreach to identify eligible households with cohabiting male–female pairs in the target age range. Interested individuals are invited to attend private screening sessions, during which study information is provided, informed consent is obtained, and baseline assessments are conducted. HIV status is determined by using records from the HDSS’s nested HIV surveillance program, which conducts home-based testing approximately every two years. At the time of recruitment, HIV status is confirmed using the most recent surveillance data and participant self-report. Individuals with unknown or discordant status are referred for voluntary testing, and those confirmed to be HIV-positive are excluded from the HIV-negative cohort.

In both cohorts, both members of the pair must meet all eligibility criteria to be enrolled. During that recruitment process, potential participants attend private screening sessions where detailed study information is provided, followed by consent procedures. Upon enrollment, participants complete baseline assessments, including anthropometric measurements, behavioral questionnaires, and allocation of wearable devices for 12 months of continuous physiological and environmental monitoring.

While recruitment settings differ—clinic-based for the HIV-positive cohort and community-based for the HIV-negative cohort—these approaches are selected for ethical, logistical, and epidemiological reasons. Clinic-based recruitment ensures privacy, accurate linkage to clinical data, and respect for disclosure practices, while HDSS-based recruitment provides efficient population-based sampling of HIV-negative individuals. These differences reflect real-world access pathways and enhance the external validity of the study.

To promote comparability, calendar–month alignment is used to match seasonal exposures across cohorts. Additionally, identical eligibility criteria, screening protocols, and data collection tools are applied. Potential differences introduced by recruitment context are addressed analytically through covariate adjustment, inclusion of cohort as a fixed effect in multivariable models, and sensitivity analyses.

### Overview of the study procedures

**Fig. 4:**
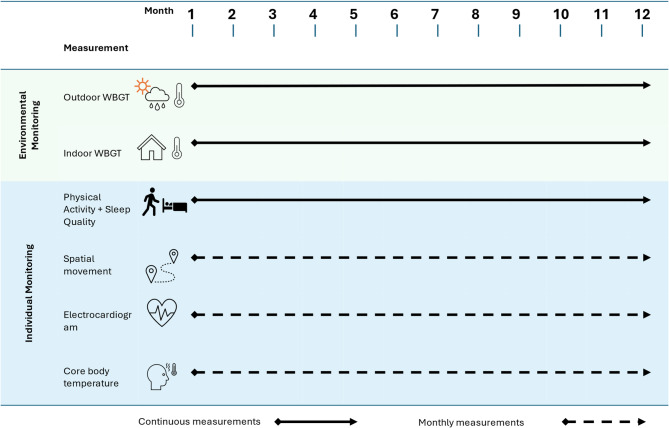
Overview of the study procedures for environmental and individual monitoring. The outdoor and indoor wet bulb globe temperature (WBGT), physical activity, and sleep quality are monitored continuously over 12 months. In addition, all participants undergo monthly 24-hour sessions to assess spatial movement, electrocardiography, and core body temperature. These periodic assessments complement continuous monitoring to capture high-resolution physiological responses

Figure 4 presents the experimental protocol for both cohorts. At study initiation, all participants undergo a baseline clinic visit for demographic and anthropometric assessments (height, weight, BMI), body composition via bioimpedance analysis, and blood pressure measurement. Each participant is provided a wrist-worn actigraphy device (ACT; GENEActiv Original, Activinsights, UK) to continuously monitor physical activity (steps, effort intensity) and sleep patterns (duration, quality) over 12 months.

The indoor WBGT is monitored using environmental data loggers (PCE-WB 20SD) placed in the sleeping room of each enrolled male–female pair by the study staff. Outdoor conditions—temperature, humidity, wind speed, and solar radiation—are continuously recorded using an OTT MetSystems automatic weather station (OTT netDL datalogger-500 series) already installed in the study area. WBGT is measured directly indoors and estimated outdoors using the Carter et al. formula [71]. Both the indoor and outdoor parameters are tracked continuously over 12 months, with quarterly sensor calibration to ensure data quality.

In addition to continuous measurements, participants undergo periodic assessments every 28 days. During each cycle, they wear additional devices for 24-hour monitoring: a head-worn core body temperature (CBT) sensor, a chest-worn electrocardiography (ECG) device, and a geo-positioning system (GPS) datalogger to track movement. This setup allows for integrated assessment of PSI components (HR and CBT) and spatial mapping of physiological strain in the 24 hours preceding the monthly clinic visit. At each visit, participants also complete a short questionnaire covering thermal comfort, work activity, and heat-related symptoms.

Data from all devices and environmental sensors are downloaded at each visit and securely stored in the study database. Device clocks are synchronized to align multi-source data accurately. Participants are encouraged to follow their usual routines, including farming, to capture representative exposure and workload patterns. The study team conducts regular checks for device performance and adherence. Any issues are addressed promptly, including device replacement where needed.

### Study instruments

**Fig. 5:**
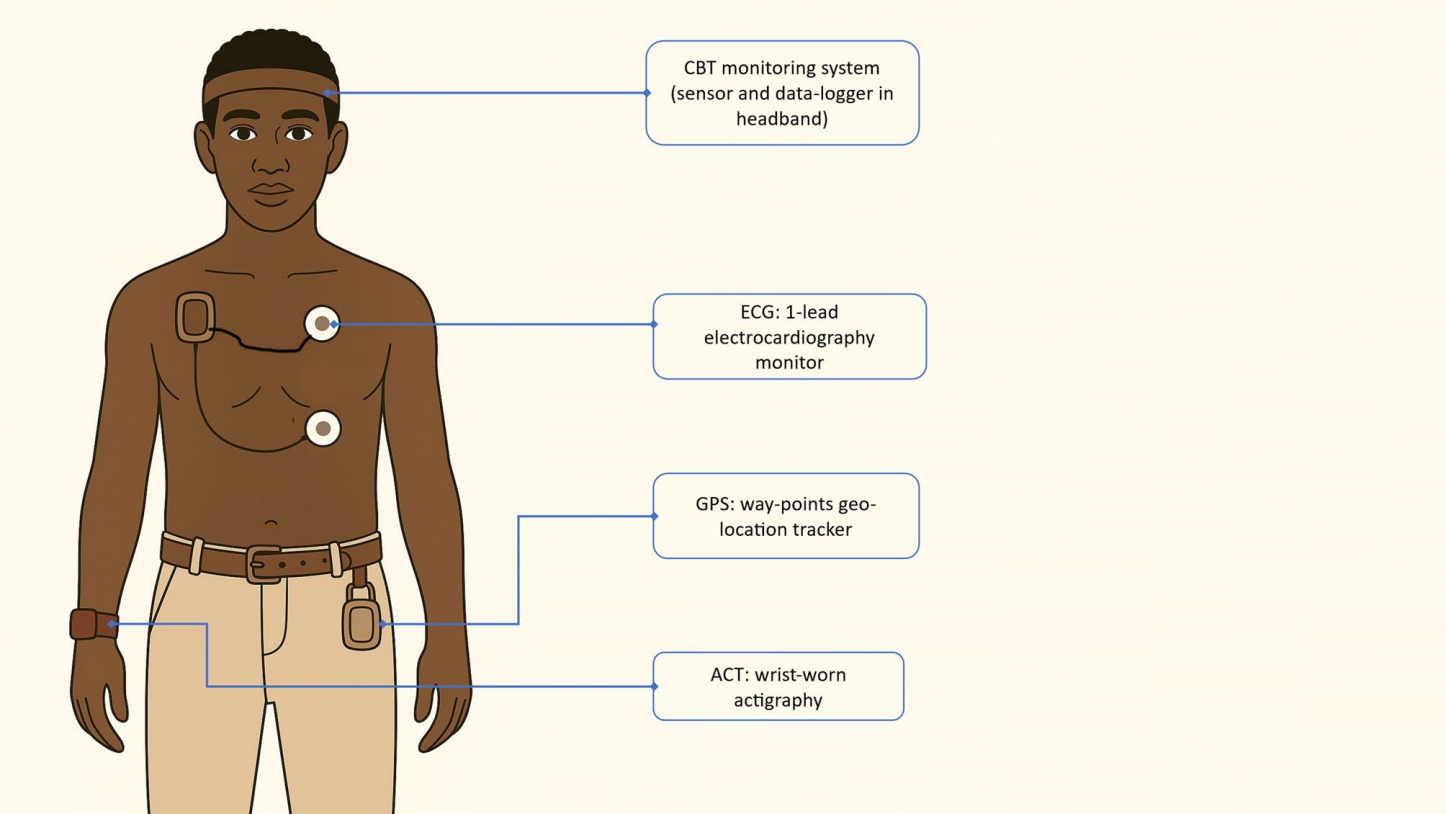
Placement of wearable devices for personal monitoring. Illustration of wearable device placement for physiological and behavioural monitoring. Devices include: a head-worn core body temperature (CBT) sensor and data logger, a 1-lead chest-worn electrocardiography (ECG) monitor, a wrist-worn actigraphy device (ACT), and a GPS waypoints tracker worn at the waist. These instruments collectively capture thermal strain, cardiovascular strain,, physical activity, sleep, and spatial movement patterns

The study employs research-grade wearables, environmental sensors, and clinical instruments. Detailed descriptions of these devices—including their technical specifications and measurement capabilities—are available in a previous publication [61] and are not repeated here. Device placement for participants is illustrated in Fig. 5.

#### Actigraphy devices (GENEActiv original, activinsights, UK)

Participants wear GENEActiv devices on the non-dominant wrist continuously to monitor physical activity, sleep, light exposure, and skin temperature. The device records triaxial acceleration at 100 Hz (in gravity units), ambient light every second (in lux), and skin temperature every 30 s (in °C) [72]. Placement on the non-dominant wrist minimizes movement artefacts, reduces device damage during routine tasks, and standardizes data collection. The device is water-resistant (IP67-rated) and remains worn during daily activities, including bathing.

Raw data are processed using the *GGIR* package in R, which calibrates acceleration, detects non-wear periods, and aggregates data into epochs [73]. Physical activity is quantified using the Euclidean Norm Minus One (ENMO), with intensity levels (light, moderate, vigorous) classified via validated cut-points. Sleep is estimated using an angle-change algorithm that identifies sustained low-movement periods with minimal posture variation, aided by light and temperature data to determine onset, offset, and duration. Implausible values are flagged and excluded. Continuous wear over 12 months provides high-resolution, real-world data on daily activity and sleep behaviour.

#### Core body temperature sensors (Tcore sensor, Dräger, Germany)

A validated Tcore sensor worn as a headband around the forehead is used to measure core body temperature (CBT) [74]. The device records data at a sampling rate of 0.0167 Hz (one data point per minute) during the 24-hour assessments, conducted monthly. Continuous CBT monitoring provides insight into thermal strain, a critical indicator of heat stress. Data collection occurs the day before the participants’ monthly clinic visits. Values outside the normal range (e.g., below 35 °C or above 42 °C) will be flagged and excluded during post-processing to ensure data quality.

#### Electrocardiography devices (Faros 180, Bittium, Finland)

Participants also undergo monthly 24-hour electrocardiography (ECG) monitoring using the Faros 180 device (Bittium, Finland), a lightweight, single-lead chest-worn recorder designed for continuous ambulatory use [75]. The device is attached using three adhesive electrodes placed in a standard thoracic configuration to ensure stable signal acquisition throughout daily activities, including physical labor.

The ECG signal is recorded at a sampling rate of 250 Hz, providing high-resolution data on cardiac electrical activity. The data will be processed using *NeuroKit2*, an open-source Python package for physiological signal analysis [76]. The signal will be cleaned and R-peaks identified, from which heart rate (HR) and heart rate variability (HRV) metrics will be derived. HR will be calculated as beats per minute, while HRV will include time-domain features such as the root mean square of successive differences (RMSSD) and the standard deviation of NN intervals (SDNN), frequency-domain measures such as low- and high-frequency power (LF, HF), and nonlinear indices derived from Poincaré plots (SD1, SD2).

These cardiac measures will be used to assess cardiovascular strain and, in combination with core body temperature (CBT), compute the Physiological Strain Index (PSI). Implausible or artefactual segments—such as extreme heart rates, flatlines, or noise from poor electrode contact- will be identified and excluded during preprocessing to ensure data quality.

#### GPS loggers (GP-102 G-Porter GPS logger, Renkforce, Germany)

Participants are also provided a Renkforce GP-102 G-Porter GPS logger at the waist, attached to a belt or clothing, during each 24-hour monthly monitoring session. The device records geospatial coordinates at a sampling rate of 1 Hz (one location point per second), enabling high-resolution tracking of movement throughout the day.

To classify time spent in different environments, each GPS waypoint will be matched to predefined geographic boundaries for the participant’s home and farm, which were mapped during a prior pilot study using handheld GPS units. A spatial overlay algorithm will be used to assign each point to one of three location categories: **home**, **farm**, or **elsewhere**. Waypoints falling outside all known boundaries will be labeled as “elsewhere” but retained for analysis unless clearly erroneous (e.g., outside the country due to signal error). From these data, the metrics derived will include total distance travelled, time spent in each location, and estimated farm work start and end times.

#### Automatic weather station

An automatic weather station, centrally located in the study area, records environmental data every 15 min, including air temperature, relative humidity, wind speed, solar radiation, and rainfall. These variables are used to estimate outdoor WBGT as a composite measure of environmental heat exposure [71, 77].

#### Indoor environmental data loggers (PCE-WB 20SD, PCE Deutschland GmbH)

Each participant household is equipped with a PCE-WB 20SD data logger, which records indoor WBGT every 10 min. The device directly measures the air temperature, relative humidity, and globe temperature. Logged values are downloaded periodically and aligned with participant-level physiological and behavioural data, providing continuous insight into heat exposure during home-based activities, including rest and sleep. The indoor WBGT is calculated as.

$$\:{\text{W}\text{B}\text{G}\text{T}}_{\text{i}\text{n}\text{d}\text{o}\text{o}\text{r}}=0.7\cdot\:{T}_{wb}+0.3\cdot\:{T}_{g}$$

- *T*_*wb*_ is the natural wet bulb temperature (influenced by humidity and airflow).
- *T*_*g*_ is the globe temperature (captures radiant heat load).

This is the ISO 7243 formula used for shaded or indoor environments to assess thermal risk without direct solar radiation.

#### Anthropometric and bioimpedance instruments

##### Stadiometer and digital scale

Participants’ height (in centimetres) and weights (in kilograms) are measured at baseline and during monthly follow-up visits to track changes in body mass index (BMI, kg/m²). Height is measured using a wall-mounted stadiometer, and weight is recorded using a calibrated digital scale, ensuring precise anthropometric data.

##### Bioimpedance analyzer (BIA 101, AKERN, Italy)

Body composition is assessed monthly using bioimpedance analysis (BIA), with electrodes placed on the hand and foot according to the manufacturer and literature guidelines [78, 79]. This device measures the body’s resistivity and reactance to a low electrical current, which is used to estimate total body water (TBW) and fat mass (FM) through an equation validated for populations geographically and ethnically closest to the study cohort [80]. Monitoring TBW and FM over time will provide insights into hydration status and changes in body composition, both of which are critical for understanding how body composition influences individuals’ physiological response to heat stress.

##### Digital sphygmomanometer (Omron JPN 600)

Blood pressure is measured monthly using a digital sphygmomanometer on the left arm, following standard protocols of having the participant seated and rested prior to measurement. This allows for the monitoring of cardiovascular health throughout the study.

#### Questionnaires

All questionnaires are administered using the *Survey Solutions* platform, developed by the World Bank for **Computer-Assisted Personal Interviews (CAPI)** [81]. Data was collected at baseline and continues monthly during follow-up visits to capture demographic information, thermal comfort, symptoms of heat-related illness (HRI), and details about participants’ work patterns. The use of CAPI ensures accurate and efficient data entry, with immediate verification of responses for quality control.

### Data management

#### Data collection and synchronization

Data from wearable and environmental devices are downloaded monthly using manufacturer-provided software (e.g., GENEActiv PC, CoreTemp Explorer, FAROS Manager). Devices are time-synchronized before each deployment to ensure alignment across data streams.

The ***Survey Solutions*** platform incorporates strong data protection features, including user authentication, role-based access control, encrypted data transmission, and secure centralized storage. Survey data are automatically uploaded to the cloud upon synchronization and stored on secure servers maintained by *Survey Solutions*.

Physiological, behavioral, and environmental datasets will be aligned using a custom-built R-based integration script [82], which merges time-stamped observations into comprehensive participant-level datasets at hourly and daily resolutions. This enables accurate correlation between environmental exposures (e.g., WBGT) and outcomes such as heart rate, core body temperature, moderate-to-vigorous-physical-activity (MVPA), and sleep quality.

#### Data quality assurance

All wearable devices and environmental sensors are calibrated before deployment and periodically during the study. Field staff conduct monthly device checks, and participants are trained in proper device use and charging. During follow-up visits, data from wearables are reviewed for completeness. Malfunctioning devices are replaced as soon as possible to minimize monitoring gaps. Data missing at random will be excluded during analysis, while structured missingness (e.g., due to device failure) will be addressed using **model-based imputation** or sensitivity analyses. During post-download validation checks, implausible or non-biological readings (e.g., HR > 220 bpm, CBT < 33 °C or > 42 °C) are flagged and excluded.

#### Data storage and security

Sensor and wearable data are stored in protected folders on a password-secured study laptop. This data is backed up in **two secure**, **encrypted external hard drives** and regularly uploaded to a **secure institutional server** with restricted access. All storage systems comply with Kenya’s Data Protection Act (2019), the Health Act (2017), the National Commission for Science, Technology and Innovation (NACOSTI) guidelines, and international standards such as the European Union General Data Protection Regulation (GDPR).

The questionnaire data stored on the *Survey Solutions* platform remain encrypted in transit and at rest. Personally identifiable information (e.g., names, addresses) are stored separately from study data, and all analysis will be conducted using de-identified datasets with unique participant identifiers (IDs) .

#### Data cleaning and documentation

After data collection, all datasets will be cleaned to remove duplicates, align time stamps, and standardize variable names and formats. Variables will be organized by participant ID with associated hourly or nightly timestamps. A codebook will be developed to describe variable definitions, data sources, processing steps, and units of measurement to facilitate reproducibility and clear interpretation.

#### Data sharing and archiving

Deidentified data will be made available to external researchers upon request, subject to Institutional Review Board (IRB) approval and national regulations. Personal identifiers will not be shared. The final datasets will be archived for at least 10 years using institutional secure storage systems and deposited in global research repositories such as those listed in *re3data.org* to promote transparency and future research use.

### Statistical analysis

All analyses will be conducted using R (version 4.3.1). The statistical plan includes descriptive summaries, derivation of exposure and outcome metrics, and inferential modeling using generalized additive mixed models (GAMMs) and causal mediation analysis.

#### Descriptive analysis

Descriptive statistics will be used to summarize baseline characteristics (e.g., age, sex, BMI, HIV status, and ART duration). Means (standard deviations) or medians (interquartile ranges) will be reported for continuous variables, and frequencies (percentages) will be reported for categorical variables. Comparisons between HIV-positive and HIV-negative participants, and between sexes, will be assessed using t-tests or Wilcoxon rank-sum tests for continuous variables and Chi-square or Fisher’s exact tests for categorical variables. Seasonal differences in indoor and outdoor WBGT will be examined using one-way ANOVA.

#### Derived variables

In all the equations below, $\:i$ represents the individual participant, and $\:t$ represents the hour of observation (used for hourly physiological and activity data. The subscript $\:h\left(i\right)$ refers to the household to which individual $\:i$ belongs. Subscripts such as $\:\text{r}\text{e}\text{s}\text{t}$ and $\:0$ (e.g., $\:H{R}_{\text{r}\text{e}\text{s}\text{t}}$, $\:{T}_{c,0}$) refer to the baseline or resting values, while $\:\text{m}\text{a}\text{x}$ (e.g., $\:H{R}_{\text{m}\text{a}\text{x}}$) refers to estimated maximum physiological capacity. Variables with subscripts such as $\:H{R}_{it}$, $\:{\text{W}\text{B}\text{G}\text{T}}_{\text{e}\text{f}\text{f},it}$, and $\:{\text{W}\text{o}\text{r}\text{k}\text{C}\text{a}\text{p}}_{it}$ vary by individual and hour, while terms such as $\:{\text{M}\text{V}\text{P}\text{A}}_{\text{r}\text{e}\text{f},i}$ are participant-specific but time-invariant.

We will derive the following:

*Physiological strain index (PSI):*

$$\:{\text{PSI}}_{it}=5\cdot\:\frac{{T}_{c,it}-{T}_{c,0}}{39.5-{T}_{c,0}}+5\cdot\:\frac{H{R}_{it}-H{R}_{0}}{H{R}_{\text{max}}-H{R}_{0}},$$

where:

- *T*_*c, it*_ and *HR*_*it*_ are the core body temperature and heart rate at time *t* for individual *i*,
- *T*_*c*,0_ and *HR*_0_ are the resting core temperature and resting heart rate,
- *HR*_max_ = 208 − 0.7 · Age_*i*_ is the maximum heart rate of individual *i*, computed using the Tanaka formula [83].

*Percent heart rate reserve (%HRR):*

$$\:\%HR{R}_{it}=100\cdot\:\frac{H{R}_{it}-H{R}_{\text{rest}}}{H{R}_{\text{max}}-H{R}_{\text{rest}}}$$

where:

- *HR*_*it*_ is the heart rate of individual *i* at time *t*,
- *HR*_rest_ is the resting heart rate of individual *i*,
- *HR*_max_ = 220 − Age_*i*_ is the maximum heart rate of individual *i*, computed using the Tanaka formula [83].

The percent HRR expresses cardiovascular effort relative to an individual’s capacity.

*Effective WBGT:*

$$ \begin{gathered} \:{\text{WBGT}}_{{{\text{eff}},it}} = P_{{{\text{home}},it}} \cdot \:{\text{WBGT}}_{{{\text{indoor}},it}} + P_{{{\text{farm}},it}} \hfill \\ \quad \cdot \:{\text{WBGT}}_{{{\text{outdoor}},t}} + P_{{{\text{other}},it}} \cdot \:{\text{WBGT}}_{{{\text{outdoor}},t}} . \hfill \\ \end{gathered} $$

where:

- P_*home,it*_, P_*farm,it*_, P_*other,it*_ represent the proportion of time interval *t* an individual *i* spends in each location, i.e. home, farm, elsewhere, respectively.
- WBGT_indoor, it_ is WBGT measured at home (mean) of individual i at time interval *t*.
- WBGT_outdoor, t_ is WBGT estimated from the weather station at time interval *t*.

The effective WBGT is a time-weighted estimate of individual heat exposure based on GPS-derived location. It is used to reflect true exposure across multiple microenvironments.

*Relative work capacity:*

$$\:{\text{WorkCap}}_{it}=100\cdot\:\frac{{\text{MVPA}}_{it}}{{\text{MVPA}}_{\text{ref},i}},$$

where:

- MVPA_*it*_ is the duration of moderate-to-vigorous physical activity by individual *i* at time interval *t*,
- MVPA_ref,*i*_ is the individual *i* average MVPA duration under thermoneutral conditions (WBGT 24–26 °C).

Relative work capacity represents the percentage of expected activity under heat stress compared with an individual’s baseline under ideal thermal conditions.

Sleep metrics (total sleep time, sleep efficiency, sleep onset latency, and wake after sleep onset) will be derived using the *GGIR* package from raw actimetry [73].

#### Adjustment for circadian, diurnal, and seasonal variation

We will adjust for multiple time-linked rhythms that may confound heat–health associations. The circadian rhythms of core body temperature, heart rate, and sleep drive will be controlled using a cyclic spline for hour of the day in all models using hourly data. This approach models the endogenous circadian ( ~ 24-hour) biological cycle and helps distinguish physiological strain due to internal circadian regulation from that due to environmental heat load. It will also capture diurnal patterns in work and activity timing. Seasonal variation in WBGT, driven by rainfall and humidity, will be modelled as a continuous exposure using WBGT itself. Agricultural activity patterns tied to farming seasons will be explored descriptively and incorporated into subgroup and stratified analyses as needed.

#### Modeling strategy

We will fit GAMMs to model nonlinear relationships between WBGT and the study outcomes, adjusting for demographic and physiological covariates and nested clustering. Each model will include:

- A spline $\:{f}_{1}\left({\text{WBGT}}_{\text{eff},it}\right)$ to capture non-linear exposure-response.
- A cyclic spline $\:{f}_{2}\left({\text{Hour}}_{t}\right)$ to adjust for time-of-day.
- Fixed effects for HIV status, sex, age, and percent fat mass.
- Interaction terms (e.g., $\:{\text{WBGT}}_{\text{eff},it}\times\:{\text{HIV}}_{i}$) to assess moderation.
- Random intercepts for individuals ($\:{u}_{i}$) and households ($\:{v}_{h\left(i\right)}$).

For example, the model for daytime work capacity will be:

$$ \begin{gathered} {\text{WorkCap}}_{{it}} = \beta \:_{0} + f_{1} \left( {{\text{WBGT}}_{{{\text{eff}},it}} } \right) + f_{2} \left( {{\text{Hour}}_{t} } \right) \hfill \\ \quad + \beta \:_{1} \cdot \:{\text{HIV}}_{i} + \beta \:_{2} \cdot \:{\text{Sex}}_{i} + \beta \:_{3} \cdot \:{\text{FatMass}}_{i} \hfill \\ \quad + \beta \:_{4} \cdot \:{\text{Age}}_{i} + \gamma \: \cdot \:\left( {{\text{WBGT}}_{{{\text{eff}},it}} \times \:{\text{HIV}}_{i} } \right) \hfill \\ \quad + u_{i} + v_{{h\left( i \right)}} + \:_{{it}} \hfill \\ \end{gathered} $$

Each $\:\beta\:$ represents the main effect of the corresponding covariate, while $\:\gamma\:$ captures moderation by HIV status.

Analogous models will be fitted for the PSI, %HRR, and sleep metrics.

While our primary modelling strategy involves generalized additive mixed models (GAMMs) to account for potential nonlinear relationships, we will also evaluate alternative modelling approaches (e.g., linear mixed-effects models or generalized estimating equations) on the basis of model diagnostics, parsimony, and goodness-of-fit criteria. The final choice of model will be guided by empirical performance and theoretical appropriateness for each outcome.

#### Causal mediation analysis

On the intensive 24-hour monitoring days, we will assess whether physiological strain mediates the effect of WBGT on work capacity or sleep efficiency. The mediation framework includes the following:

*Mediator model:*

$$\:{\text{PSI}}_{it}={\alpha\:}_{0}+f\left({\text{WBGT}}_{\text{eff},it}\right)+\text{covariates}+{u}_{i}$$

*Outcome model:*

$$ \begin{gathered} \:{\text{WorkCap}}_{{it}} = \beta \:_{0} + f\left( {{\text{WBGT}}_{{{\text{eff}},it}} } \right) \hfill \\ \quad + \beta \:_{1} \cdot \:{\text{PSI}}_{{it}} + {\text{covariates}} + u_{i} \hfill \\ \end{gathered} $$

We will estimate the average causal mediation effect (ACME), average direct effect (ADE), and proportion mediated using bootstrap confidence intervals.

#### Sensitivity analyses

We will examine alternative WBGT metrics (e.g., daily maximum, minimum, and 7-day cumulative), use splines to identify thresholds, and compare alternative model specifications including linear mixed-effects models and generalized estimating equations. Subgroup analyses will be conducted by HIV status, sex, and fat mass tertile. MVPA models will be restricted to GPS-classified farming periods to assess occupational heat exposure. Propensity score matching will be used to adjust for baseline differences between HIV-positive and HIV-negative cohorts.

#### Model validation

Model validation will be conducted through residual diagnostics, including residual-versus-fitted plots, quantile–quantile plots, and checks for temporal autocorrelation. We will assess the complexity of smooth terms via estimated degrees of freedom and evaluate concurvity to detect multicollinearity among spline terms. Competing model structures (e.g., with and without interaction terms or splines) will be compared using Akaike Information Criterion (AIC) and graphical assessments of fit.

### Ethics and dissemination

**Informed consent:** All participants will provide written informed consent prior to enrollment. Study staff will offer a detailed explanation of the study’s purpose, procedures, potential risks, and anticipated benefits. Participants will be informed of their right to withdraw at any time without consequence. No individual will be enrolled without signed consent.

**Ethical approval:** Ethical approval has been obtained from the institutional review boards of Charité – Universitätsmedizin Berlin (EA1/044/22) and the Kenya Medical Research Institute (KEMRI/SERU/CGHR/03-09-431/4640).

**Privacy and confidentiality:** To protect participant confidentiality, all data are pseudo-anonymized and stored on password-protected servers with access restricted to authorized personnel. Data handling procedures comply with relevant data protection regulations and institutional policies.

**Dissemination plan:** Engagement and dissemination are planned at multiple stages. Prior to data collection, approval was sought from local health authorities, including the Siaya County Department of Health. Ongoing community sensitization is conducted through baraza (public forums) and government staff workshops. Throughout the study, continuous engagement is maintained via Community Advisory Board (CAB) meetings, and quarterly joint co-audits with County Health Management Teams (CHMTs) support transparency and responsiveness. Upon study completion, preliminary findings will be shared with local stakeholders to validate interpretation and guide next steps. Policy briefs will be prepared for relevant ministries (Health, Environment), while peer-reviewed publications and international presentations will ensure broader dissemination. De-identified datasets may be made available for secondary analyses upon reasonable request and subject to ethical approval, fostering ongoing research on climate-health interactions in vulnerable populations.

## Discussion

This study protocol presents a novel and comprehensive investigation into the impacts of environmental heat on labour capacity in rural sub-Saharan Africa, focusing on smallholder agricultural workers in Siaya County, Kenya. By integrating continuous longitudinal monitoring of physiological responses, environmental exposure, and behavioral patterns, the study aims to uncover how chronic HIV infection may amplify heat vulnerability and reduce work capacity. The 12-month design captures seasonal variation, enabling a deeper understanding of fluctuations in exposure and strain across different climatic conditions and agricultural calendars.

A particular strength of this study is its focus on a population that is highly vulnerable to climate and health risks and is largely underrepresented in occupational health research. The inclusion of both HIV-positive and HIV-negative participants, as well as both men and women, allows for direct comparison of physiological strain under real-world working conditions and provides an evidence base for sex- and HIV-sensitive adaptation strategies. The use of research-grade wearables and automated environmental sensors enhances ecological validity, supporting objective assessment of exposure–response relationships that can inform policy and practice.

However, the study also faces several limitations. It is geographically restricted to a single rural setting, and although generalizability to other agroecological zones may be limited, the protocol provides a replicable model that can be adapted for use across diverse contexts in sub-Saharan Africa. Seasonal matching of HIV-positive and HIV-negative cohorts by calendar month helps mitigate confounding by weather.Additionally, potential selection bias remains if individuals with lower fitness or greater illness opt out of participation.

Maintaining device functionality and participant retention across a 12-month study period in a rural environment is challenging and may lead to missing data. These issues will be addressed analytically using sensitivity analyses and appropriate imputation methods. The combination of wearable sensor data and self-reported information balances objectivity with contextual insight, although recall bias and behavioral adaptations (such as avoiding peak heat) may affect the interpretation of exposure–response dynamics. These are addressed by linking GPS data with self-reports and accounting for shared household influences through random intercepts in statistical models.

This study is expected to yield critical evidence to inform public health strategies, occupational safety standards, and climate adaptation initiatives. The findings can support targeted interventions such as tailored work-rest cycles, hydration support, or differentiated thresholds for heat warnings based on HIV status and sex. In doing so, this research contributes to equitable climate-health adaptation by centering the needs of marginalized agricultural populations affected by intersecting vulnerabilities.

Beyond immediate applications, this protocol lays the groundwork for future research on chronic disease and environmental stress interactions. Its methods may be extended to study diabetes, cardiovascular disease, or other conditions influencing thermoregulation. The rich, longitudinal dataset produced will also enable predictive modeling of labor outcomes under future climate scenarios, providing an evidence base for health systems, policymakers, and agricultural stakeholders to plan more resilient livelihoods.

In conclusion, this protocol advances an interdisciplinary, equity-focused approach to understanding and mitigating the health impacts of climate change in sub-Saharan Africa, where heat exposure and HIV burden converge to shape labor, productivity, and well-being.

## Supplementary Information

Below is the link to the electronic supplementary material.

Supplementary material 1 (DOCX 40 kb)

## Data Availability

Data are not available at this stage because analysis is still ongoing. Upon publication of the main results, de-identified datasets may be shared by the lead author (DK) upon reasonable request, subject to approval by the Kenya Medical Research Institute Scientific and Ethics Review Unit and compliance with institutional and ethical data protection protocols.
