# Supplementary material for: Burden of heat stress on residual work capacity among farmers living with chronic HIV in Siaya county, Kenya: a longitudinal observational study protocol
Source: BMC Public Health. 2025 Aug 27;25:2940. doi: 10.1186/s12889-025-24373-w (PMC12382208; doi:10.1186/s12889-025-24373-w)
Supplement: Supplementary file 1 — Supplementary material 1 (DOCX 40 kb) [file 12889_2025_24373_MOESM1_ESM.docx]

# SUPPLEMENTARY MATERIAL: STUDY QUESTIONNAIRE

The study questionnaire is a structured instrument designed to capture multi-dimensional data on environmental heat exposure, physiological responses, and functional health outcomes among agricultural workers. It includes modules on demographics, health history, anthropometry, sleep and recovery, and recent work and heat exposure, as well as detailed sections on sensor use and device compliance. For female participants, a women’s health module captures reproductive history and menstrual impacts. The questionnaire also incorporates the 18-item Heat Strain Symptom Index (HSSI) to assess real-time subjective symptoms of heat stress. Data are collected through computer-assisted personal interviews (CAPI) at monthly follow-up visits, enabling linkage with continuous sensor data and environmental measurements.

**Modules:**

1. **Demographics (Administered at Baseline Only)**
2. **Health History (Administered at Baseline Only)**
3. **Anthropometry & Clinical Data**
4. **Sensor Use & Device Management**
5. **Heat Exposure at Work**
6. **Home Heat Exposure**
7. **Work History (2-Year Recall- Administered at Baseline Only)**
8. **Women’s Health** *(conditional on sex)*
9. **Sleep & Recovery**
10. **Symptom Diary**
11. **Heat Strain Symptom Index (HSSI)**

**DEMOGRAPHICS MODULE (Administered at Baseline Only)**

**1. Participant ID**
*Response type: Open text*
**Response:**  __________

**2. Name**
*Response type: Open text*
**Response:**  __________

**3. Village**
*Response type: Open text*
**Response:**  __________

**4. Age (in completed years)**
*Response type: Numeric*
**Response:**  __________

**5. Sex**
*Response type: Single select*
**Response:**

- (◯) Male
- (◯) Female
- (◯) Other

**6. Marital Status**
*Response type: Single select*
**Response:**

- (◯) Single (never married)
- (◯) Married
- (◯) Separated
- (◯) Divorced
- (◯) Widowed
- (◯) Cohabiting, unmarried

**7. What is the highest level of education you have completed?**
*Response type: Single select*
**Response:**

- (◯) No formal schooling
- (◯) Primary incomplete
- (◯) Primary complete
- (◯) Secondary incomplete
- (◯) Secondary complete
- (◯) Post-secondary (college, university, vocational)
- (◯) Other: _________

**8. Are you able to read and write in any of the following languages?**
*Response type: Multi select*
**Instruction:** Select all that apply.
**Response:**

- Kiswahili
- English
- Dholuo
- None

**9. Do you have access to a mobile phone (shared or personal)?**
*Response type: Single select*
**Response:**

- (◯) Yes
- (◯) No

**10. How many people currently live in your household (including yourself)?**
*Response type: Numeric*
**Response:**  __________

**11. What is your main occupation?**
*Response type: Open text*
**Response:**  __________

**12. How long have you lived in this household? (in months or years)**
*Response type: Open text / Numeric*
**Response:**  __________

**13. Is your household currently registered in the KEMRI/CDC HDSS system?**
*Response type: Single select*
**Response:**

- (◯) Yes
- (◯) No
- (◯) Don’t know

**HEALTH HISTORY MODULE (Administered at Baseline Only)**

**1. Do you currently smoke any tobacco products (e.g., cigarettes, hand-rolled tobacco, pipe)?**
*Response type: Single select*
**Response:**

- (◯) Yes
- (◯) No

**2. In the past 12 months, have you consumed alcohol?**
*Response type: Single select*
**Response:**

- (◯) Yes
- (◯) No

**3. Are you currently pregnant?** *(women only)*
*Response type: Single select*
**Skip pattern:** Asked only if sex = Female
**Response:**

- (◯) Yes
- (◯) No
- (◯) Don’t know

**4. Have you ever been diagnosed by a health professional with any of the following conditions?**
*Response type: Multi select*
**Instruction:** Select all that apply.
**Response:**

- Diabetes
- High blood pressure (hypertension)
- Asthma or other chronic respiratory disease
- Heart disease
- Stroke
- HIV
- Tuberculosis
- Other chronic condition: _________
- None

**5. If diagnosed with any of the above, are you currently on treatment?**
*Response type: Single select*
**Response:**

- (◯) Yes
- (◯) No
- (◯) Not applicable

**6. Do you experience any difficulties performing daily activities due to a health condition (e.g., walking, lifting, standing, working)?**
*Response type: Single select*
**Response:**

- (◯) Yes
- (◯) No

**ANTHROPOMETRY & CLINICAL DATA MODULE**

**1. What is the participant’s height (in centimeters)?**
*Response type: Numeric (measured)*
**Response:**  __________ cm

**2. What is the participant’s weight (in kilograms)?**
*Response type: Numeric (measured)*
**Response:**  __________ kg

**3. What is the participant’s Body Mass Index (BMI)?**
*Response type: Derived from weight and height*
**Formula:** BMI = weight (kg) ÷ [height (m)]²
**Response:**  __________

**4. What is the participant’s blood pressure?**
*Response type: Numeric (measured, 2 readings)*
**Response:**

- Systolic (mmHg): __________
- Diastolic (mmHg): __________

**5. What is the participant’s resting heart rate (beats per minute)?**
*Response type: Numeric (measured)*
**Response:**  __________ bpm

**6. What is the participant’s body composition based on Bioelectrical Impedance Analysis (BIA)?**
*Response type: Numeric (measured)*
**Response:**

- Resistance (Ω): __________
- Reactance (Ω): __________
- Fat mass (%): __________ *(calculated or device-provided)*

**7. When was the last meal or drink (except water) consumed before BIA?**
*Response type: Open text or categorical*
**Response:**  __________
*(Optional: include time elapsed or standardize for fasting)*

**8. Time of day measurement was taken:**
*Response type: Time field or open text*
**Response:**  __________

**SENSOR USE & DEVICE MANAGEMENT MODULE**

**1. Was the participant issued all required devices?**
*Response type: Multi select*
**Instruction:** Tick all that were provided.
**Response:**

- Wrist-worn activity monitor (actigraphy)
- Chest ECG patch
- Core body temperature sensor
- GPS tracker
- Indoor WBGT logger (for home)
- None

**2. Was the participant trained on how to use each device?**
*Response type: Single select*
**Response:**

- (◯) Yes
- (◯) No

**3. Did the participant confirm understanding of how to wear and charge the devices?**
*Response type: Single select*
**Response:**

- (◯) Yes
- (◯) No

**4. Was the participant given written instructions or visual guides?**
*Response type: Single select*
**Response:**

- (◯) Yes
- (◯) No

**5. Were there any issues observed or reported with device fitting or comfort?**
*Response type: Single select*
**Response:**

- (◯) Yes
- (◯) No
  **If Yes, specify issue:**
  *Response type: Open text*
  **Response:**  __________

**6. Was the indoor WBGT logger placed in the household?**
*Response type: Single select*
**Response:**

- (◯) Yes
- (◯) No
  **If No, explain why:**
  *Response type: Open text*
  **Response:**  __________

**7. At device return, were all devices accounted for and functioning?**
*Response type: Single select*
**Response:**

- (◯) Yes
- (◯) No
  **If No, specify missing/damaged device(s):**
  *Response type: Open text*
  **Response:**  __________

**8. Was data successfully downloaded from all devices?**
*Response type: Single select*
**Response:**

- (◯) Yes
- (◯) No
  **If No, describe issue (e.g., no data, file corrupted):**
  *Response type: Open text*
  **Response:**  __________

**HEAT EXPOSURE AT WORK MODULE**

**1. In the past 7 days, did you do any work outside the home (e.g., farming, construction, trading, etc.)?**
*Response type: Single select*
**Response:**

- (◯) Yes
- (◯) No
  **Skip pattern:** If "No", skip to **Q10** (Indoor work or heat exposure at home).

**2. What type of outdoor work did you do most often in the past week?**
*Response type: Open text*
**Response:**  __________

**3. On which days did you work outdoors?**
*Response type: Multi select*
**Instruction:** Tick all that apply.
**Response:**

- Monday
- Tuesday
- Wednesday
- Thursday
- Friday
- Saturday
- Sunday

**4. What were your approximate working hours (start and end time) on a typical day last week?**
*Response type: Open text (time range)*
**Response:**  __________ to __________

**5. Did you work outdoors during the hottest hours of the day (e.g., 11am to 3pm)?**
*Response type: Single select*
**Response:**

- (◯) Yes
- (◯) No
- (◯) Not sure

**6. Did you experience any of the following symptoms while working in the heat in the past week?**
*Response type: Multi select*
**Instruction:** Tick all that apply.
**Response:**

- Feeling very hot or flushed
- Excessive sweating
- Headache
- Dizziness or lightheadedness
- Muscle cramps
- Nausea or vomiting
- Rapid heartbeat
- Confusion or fainting
- None of the above

**7. Did heat affect your ability to work in the past 7 days?**
*Response type: Single select*
**Response:**

- (◯) Yes
- (◯) No
- (◯) Not sure
  **If Yes:**
  **7a. How did heat affect your work?**
  *Response type: Multi select*
  **Response:**
- Took more breaks
- Worked fewer hours
- Skipped work entirely
- Worked at a slower pace
- Changed clothing or location
- Other: __________

**8. Did you skip work due to heat in the past week?**
*Response type: Single select*
**Response:**

- (◯) Yes
- (◯) No

**9. What did you do to cope with the heat while working?**
*Response type: Multi select*
**Instruction:** Tick all that apply.
**Response:**

- Drank more water
- Took rest breaks
- Found shade
- Wore lighter clothing
- Changed work time to morning/evening
- Nothing
- Other: __________

**10. Do you do any indoor work (e.g., cooking, weaving, tailoring, etc.)?**
*Response type: Single select*
**Response:**

- (◯) Yes
- (◯) No
  **If Yes, continue to indoor heat exposure module.**

**HOME HEAT EXPOSURE MODULE**

**1. During the hottest months of the year, do you usually feel uncomfortable due to heat inside your home?**
*Response type: Single select*
**Response:**

- (◯) Yes
- (◯) No
- (◯) Not sure

**2. During what time of day does your home feel hottest?**
*Response type: Single select*
**Response:**

- (◯) Morning (6 am – 10 am)
- (◯) Midday (10 am – 2 pm)
- (◯) Afternoon (2 pm – 6 pm)
- (◯) Evening (6 pm – 10 pm)
- (◯) Night (10 pm – 6 am)
- (◯) All day
- (◯) Don’t know

**3. What parts of your home get hottest during the day?**
*Response type: Multi select*
**Instruction:** Tick all that apply.
**Response:**

- Bedroom
- Kitchen
- Living room
- Outside veranda
- Entire house
- Other: __________

**4. What do you usually do to cope with the heat while at home?**
*Response type: Multi select*
**Instruction:** Tick all that apply.
**Response:**

- Open doors/windows
- Move to a cooler room
- Go outside under shade
- Reduce activity
- Wet towel or cloth
- Bathe
- Drink cold water
- Nothing
- Other: __________

**5. Is there electricity in your home?**
*Response type: Single select*
**Response:**

- (◯) Yes
- (◯) No

**6. Do you have a fan in your home?**
*Response type: Single select*
**Response:**

- (◯) Yes
- (◯) No

**7. Is there any form of shading around your home (e.g., trees, roof extension)?**
*Response type: Single select*
**Response:**

- (◯) Yes
- (◯) No

**8. During the past 4 weeks, did heat in your home disturb your sleep?**
*Response type: Single select*
**Response:**

- (◯) Yes
- (◯) No
- (◯) Not sure

**9. What do you do when it is too hot to sleep inside your home?**
*Response type: Multi select*
**Response:**

- Sleep outside
- Sleep in another room
- Open more windows/doors
- Use wet cloth or water
- Remove bedding or clothing
- Nothing
- Other: __________

**WORK HISTORY (2-YEAR RECALL) MODULE- Administered only at Baseline**

**1. Have you done any income-generating or subsistence work in the past 2 years?**
*Response type: Single select*
**Response:**

- (◯) Yes
- (◯) No
  **Skip pattern:** If "No", skip to end of module.

**2. What type(s) of work have you done in the past 2 years?**
*Response type: Multi select*
**Instruction:** Tick all that apply.
**Response:**

- Farming (crop cultivation)
- Livestock keeping
- Charcoal burning
- Construction or masonry
- Fishing
- Casual labor (e.g., sugarcane cutting)
- Small-scale trading
- Brick making
- Teaching or childcare
- Domestic work
- Other: __________

**3. Was this work mostly done indoors, outdoors, or both?**
*Response type: Single select*
**Response:**

- (◯) Mostly outdoors
- (◯) Mostly indoors
- (◯) Both equally

**4. During the hot season, do you reduce or change your work activities?**
*Response type: Single select*
**Response:**

- (◯) Yes
- (◯) No
  **If Yes:**
  **4a. How do you change your work during the hot season?**
  *Response type: Multi select*
  **Response:**
- Start work earlier
- Work fewer hours
- Avoid midday sun
- Work in shaded areas
- Reduce work intensity
- Stop working temporarily
- Other: __________

**5. Have you ever lost income due to heat interfering with your ability to work?**
*Response type: Single select*
**Response:**

- (◯) Yes
- (◯) No
- (◯) Not sure

**6. Does your main occupation differ between rainy and dry seasons?**
*Response type: Single select*
**Response:**

- (◯) Yes
- (◯) No
  **If Yes:**
  **6a. Please describe the difference:**
  *Response type: Open text*
  **Response:**  __________

**7. In which months of the year do you typically work the most?**
*Response type: Multi select*
**Instruction:** Tick all applicable months.
**Response:**

- January
- February
- March
- April
- May
- June
- July
- August
- September
- October
- November
- December

**WOMEN’S HEALTH MODULE**

**(Only asked if participant is female)**
*Skip pattern: If participant is male, skip this module.*

**1. Are you currently pregnant?**
*Response type: Single select*
**Response:**

- (◯) Yes
- (◯) No
- (◯) Not sure

**2. How many children have you given birth to?**
*Response type: Numeric*
**Response:**  __________

**3. When was your last delivery?**
*Response type: Open text / Month and Year*
**Response:**  __________

**4. Are you currently using any contraceptive method?**
*Response type: Single select*
**Response:**

- (◯) Yes
- (◯) No

**5. What type of contraceptive method are you using?**
*Response type: Multi select*
**Instruction:** Tick all that apply.
**Response:**

- Oral contraceptive pills
- Injectable contraception
- Implant
- Intrauterine device (IUD)
- Male condom
- Female condom
- Tubal ligation
- Lactational amenorrhea method (LAM)
- Traditional method
- Other: __________

**6. Have you experienced menstrual periods in the last 3 months?**
*Response type: Single select*
**Response:**

- (◯) Yes
- (◯) No
- (◯) Not applicable (e.g., post-menopausal)

**7. In the past 3 months, have your periods been regular (i.e., occur at roughly the same time each month)?**
*Response type: Single select*
**Skip pattern:** Only asked if Q6 = Yes
**Response:**

- (◯) Yes
- (◯) No

**8. Do you experience any of the following symptoms during your period?**
*Response type: Multi select*
**Instruction:** Tick all that apply.
**Response:**

- Lower abdominal cramps
- Headache
- Fatigue
- Back pain
- Nausea
- Dizziness
- Heavy bleeding
- None of the above

**9. During your last period, did you stop or reduce any of the following activities because of symptoms?**
*Response type: Multi select*
**Instruction:** Tick all that apply.
**Response:**

- Farming or physical work
- Attending school or training
- Attending social events
- Caring for children or family
- None
- Other: __________

**SLEEP & RECOVERY MODULE**

**1. On a typical night in the past 7 days, what time did you usually go to sleep?**
*Response type: Time field (24-hour or AM/PM)*
**Response:**  __________

**2. On a typical morning, what time did you usually wake up?**
*Response type: Time field (24-hour or AM/PM)*
**Response:**  __________

**3. Do you usually take naps during the day?**
*Response type: Single select*
**Response:**

- (◯) Yes
- (◯) No

**4. If yes, how long do you typically nap each day?**
*Response type: Open text (minutes or hours)*
**Skip pattern:** Only if Q3 = Yes
**Response:**  __________

**5. In the past 7 days, how often did you feel well-rested after sleeping at night?**
*Response type: Single select*
**Response:**

- (◯) Every day
- (◯) Most days
- (◯) Some days
- (◯) Rarely
- (◯) Never

**6. Did you experience any of the following symptoms during the night in the past week?**
*Response type: Multi select*
**Instruction:** Tick all that apply.
**Response:**

- Waking up due to heat
- Sweating at night
- Headache
- Restlessness
- None of the above
- Other: __________

**7. What strategies did you use to sleep better on hot nights?**
*Response type: Multi select*
**Instruction:** Tick all that apply.
**Response:**

- Opened windows or doors
- Used a fan
- Took a bath before bed
- Slept outside
- Slept without bedding
- Reduced clothing
- Slept in another room
- Did nothing
- Other: __________

**SLEEP & RECOVERY MODULE**

**1. On a typical night in the past 7 days, what time did you usually go to sleep?**
*Response type: Time field (24-hour or AM/PM)*
**Response:**  __________

**2. On a typical morning, what time did you usually wake up?**
*Response type: Time field (24-hour or AM/PM)*
**Response:**  __________

**3. Do you usually take naps during the day?**
*Response type: Single select*
**Response:**

- (◯) Yes
- (◯) No

**4. If yes, how long do you typically nap each day?**
*Response type: Open text (minutes or hours)*
**Skip pattern:** Only if Q3 = Yes
**Response:**  __________

**5. In the past 7 days, how often did you feel well-rested after sleeping at night?**
*Response type: Single select*
**Response:**

- (◯) Every day
- (◯) Most days
- (◯) Some days
- (◯) Rarely
- (◯) Never

**6. Did you experience any of the following symptoms during the night in the past week?**
*Response type: Multi select*
**Instruction:** Tick all that apply.
**Response:**

- Waking up due to heat
- Sweating at night
- Headache
- Restlessness
- None of the above
- Other: __________

**7. What strategies did you use to sleep better on hot nights?**
*Response type: Multi select*
**Instruction:** Tick all that apply.
**Response:**

- Opened windows or doors
- Used a fan
- Took a bath before bed
- Slept outside
- Slept without bedding
- Reduced clothing
- Slept in another room
- Did nothing
- Other: __________

**SYMPTOM DIARY & HEAT STRAIN SYMPTOM INDEX (HSSI) MODULE**

**1. In the past 4 weeks, how often did you experience any of the following symptoms during hot weather?**
*Response type: Multiple rows of single-select responses*
**Instruction:** For each symptom, select the frequency.

| Symptom | Response Options |
| --- | --- |
| Feeling unusually tired | (◯) Never (◯) Rarely (◯) Sometimes (◯) Often (◯) Always |
| Dizziness or lightheadedness | (◯) Never (◯) Rarely (◯) Sometimes (◯) Often (◯) Always |
| Excessive sweating | (◯) Never (◯) Rarely (◯) Sometimes (◯) Often (◯) Always |
| Muscle cramps | (◯) Never (◯) Rarely (◯) Sometimes (◯) Often (◯) Always |
| Fast heartbeat while resting | (◯) Never (◯) Rarely (◯) Sometimes (◯) Often (◯) Always |
| Nausea or vomiting | (◯) Never (◯) Rarely (◯) Sometimes (◯) Often (◯) Always |
| Confusion or trouble concentrating | (◯) Never (◯) Rarely (◯) Sometimes (◯) Often (◯) Always |
| Difficulty sleeping due to heat | (◯) Never (◯) Rarely (◯) Sometimes (◯) Often (◯) Always |

**2. Did you seek medical care due to any of the above symptoms in the past 4 weeks?**
*Response type: Single select*
**Response:**

- (◯) Yes
- (◯) No

**3. Have these symptoms ever prevented you from completing your daily activities (e.g., farming, cooking, walking)?**
*Response type: Single select*
**Response:**

- (◯) Yes
- (◯) No

**4. Do you believe these symptoms were caused by exposure to heat?**
*Response type: Single select*
**Response:**

- (◯) Yes
- (◯) No
- (◯) Not sure

**HEAT STRAIN SYMPTOM INDEX (HSSI) MODULE**

**Instruction to respondent:** *Please answer the following questions based on how you feel today.*

**1. What is your feeling about the air temperature at home?**
*Response type: Single select*
**Response:**

- (◯) Very cold
- (◯) Cold
- (◯) Neutral
- (◯) Hot
- (◯) Very hot

**2. What is your current level of sweating?**
*Response type: Single select*
**Response:**

- (◯) None
- (◯) A little
- (◯) Moderate
- (◯) A lot
- (◯) Profuse

**3. How thirsty are you right now?**
*Response type: Single select*
**Response:**

- (◯) Not at all
- (◯) Slightly
- (◯) Moderately
- (◯) Very
- (◯) Extremely

**4. Do you feel dizzy or lightheaded?**
*Response type: Single select*
**Response:**

- (◯) Not at all
- (◯) Slightly
- (◯) Moderately
- (◯) Very
- (◯) Extremely

**5. How would you describe your breathing right now?**
*Response type: Single select*
**Response:**

- (◯) Very easy
- (◯) Easy
- (◯) Neutral
- (◯) Somewhat difficult
- (◯) Very difficult

**6. Are you experiencing muscle cramps right now?**
*Response type: Single select*
**Response:**

- (◯) No cramps
- (◯) Slight cramps
- (◯) Moderate cramps
- (◯) Strong cramps
- (◯) Severe cramps

**7. Are you experiencing any nausea or urge to vomit right now?**
*Response type: Single select*
**Response:**

- (◯) Not at all
- (◯) Slight
- (◯) Moderate
- (◯) Strong
- (◯) Extreme

**8. Are you experiencing a headache right now?**
*Response type: Single select*
**Response:**

- (◯) None
- (◯) Slight
- (◯) Moderate
- (◯) Strong
- (◯) Very strong

**9. Are you experiencing any fatigue or unusual tiredness?**
*Response type: Single select*
**Response:**

- (◯) None
- (◯) Slight
- (◯) Moderate
- (◯) Strong
- (◯) Very strong

**10. Are you having any difficulty concentrating or feel mentally slow?**
*Response type: Single select*
**Response:**

- (◯) Not at all
- (◯) Slight
- (◯) Moderate
- (◯) Strong
- (◯) Very strong

**11. How is your current mood?**
*Response type: Single select*
**Response:**

- (◯) Very positive
- (◯) Somewhat positive
- (◯) Neutral
- (◯) Somewhat negative
- (◯) Very negative

**12. Are you experiencing any chills or shivering?**
*Response type: Single select*
**Response:**

- (◯) Not at all
- (◯) Slight
- (◯) Moderate
- (◯) Strong
- (◯) Very strong

**13. Are you experiencing any skin flushing or redness?**
*Response type: Single select*
**Response:**

- (◯) Not at all
- (◯) Slight
- (◯) Moderate
- (◯) Strong
- (◯) Very strong

**14. Do you feel confused or disoriented?**
*Response type: Single select*
**Response:**

- (◯) Not at all
- (◯) Slightly
- (◯) Moderately
- (◯) Very
- (◯) Extremely

**15. Are you experiencing rapid heartbeat or palpitations?**
*Response type: Single select*
**Response:**

- (◯) No
- (◯) Slight
- (◯) Moderate
- (◯) Strong
- (◯) Very strong

**16. Are you currently experiencing any chest discomfort?**
*Response type: Single select*
**Response:**

- (◯) None
- (◯) Slight
- (◯) Moderate
- (◯) Strong
- (◯) Very strong

**17. Do you feel faint or like you might pass out?**
*Response type: Single select*
**Response:**

- (◯) Not at all
- (◯) Slightly
- (◯) Moderately
- (◯) Very
- (◯) Extremely

**18. Have you stopped or reduced any activity today because of heat-related symptoms?**
*Response type: Single select*
**Response:**

- (◯) No
- (◯) Yes, slightly
- (◯) Yes, moderately
- (◯) Yes, significantly
- (◯) Yes, completely stopped

**Please add here any further general comment that may be useful**
*Response Type:* Text
(A general-purpose comment box at the end of the questionnaire to record anything not covered elsewhere.)
